# MRI assessment of aortic flow in patients with pulmonary arterial hypertension in response to exercise

**DOI:** 10.1186/s12880-018-0298-9

**Published:** 2018-12-22

**Authors:** Jacob A. Macdonald, Christopher J. Franҫois, Omid Forouzan, Naomi C. Chesler, Oliver Wieben

**Affiliations:** 1 Department of Medical Physics, University of Wisconsin, 1111 Highland Avenue, Room 1005, Madison, WI 53705 USA; 2 Department of Radiology, University of Wisconsin, Madison, USA; 3 Department of Biomedical Engineering, University of Wisconsin, Madison, USA

**Keywords:** Exercise, Pulmonary arterial hypertension, Stress test, Flow, MRI

## Abstract

**Background:**

While primarily a right heart disease, pulmonary arterial hypertension (PAH) can impact left heart function and aortic flow through a shifted interventricular septum from right ventricular pressure overload and reduced left ventricular preload, among other mechanisms. In this study, we used phase contrast (PC) MRI and a modest exercise challenge to examine the effects of PAH on systemic circulation. While exercise challenges are typically performed with ultrasound in the clinic, MRI exercise studies allow for more reproducible image alignment, more accurate flow quantification, and improved tissue contrast.

**Methods:**

Six PAH patients and fifteen healthy controls (8 older age-matched, 7 younger) exercised in the magnet bore with an MRI-compatible exercise device that allowed for scanning immediately following cessation of exercise. PC scans were performed in the ascending aorta during a breath hold immediately after modest exercise to non-invasively measure stroke volume (SV), cardiac output (CO), aortic peak systolic flow (PSF), and aortic wall stiffness via relative area change (RAC).

**Results:**

Images following exercise showed mild blurring, but were high enough quality to allow for segmentation of the aorta. While SV was approximately 30% lower in PAH patients (SV_PAH,rest_ = 67 ± 16 mL; SV_PAH,stress_ = 90 ± 42 mL) than age-matched controls (SV_,older,rest_ = 93 ± 16 mL; SV_older,stress_ = 133 ± 40 mL) at both rest and following exercise, CO was similar for both groups following exercise (CO_PAH,stress_ = 10.8 ± 5.7 L/min; CO_older,stress_ = 11.8 ± 5.0 L/min). This was achieved through a compensatory increase in heart rate in the PAH subjects (74% increase as compared to 29% in age-matched controls). The PAH subjects also demonstrated reduced aortic peak systolic flow relative to the healthy controls (PSF_PAH_,_rest_ = 309 ± 52 mL/s; PSF_older_,_rest_ = 416 ± 114 mL/s; PSF_PAH_,_stress_ = 388 ± 113 mL/s; PSF_older_,_stress_ = 462 ± 176 mL/s). PAH patients and older controls demonstrated stiffer aortic walls when compared to younger controls (RAC_PAH,rest_ = 0.15 ± 0.05; RAC_older,rest_ = 0.17 ± 0.05; RAC_young,rest_ = 0.28 ± 0.08).

**Conclusions:**

PC MRI following a modest exercise challenge was capable of detecting differences in left heart dynamics likely induced from PAH. These results demonstrated that PAH can have a significant influence on systemic flow, even when the patient has no prior left heart disease. Image quantification following exercise could likely be improved in future studies through the implementation of free-breathing or real-time MRI acquisitions.

**Trial registration:**

Retrospectively registered on 02/26/2018 (TRN:NCT03523910).

## Background

It is well established that stress tests can reveal important diagnostic information beyond that seen with tests performed at a resting heart rate [1–4], such as ischemia, increased ventricular pressures, valvular regurgitation, and deficiencies in recovery to a resting state. In clinical settings, exercise tests are commonly performed with an exercise paradigm on a treadmill or stationary bicycle followed by echocardiography [3] or single photon emission computed tomography (SPECT) image acquisitions [5].

Cardiopulmonary exercise testing is increasingly used to assess disease progression in patients with pulmonary hypertension (PH) [6–8]. PH is characterized by an increase in blood pressure in the pulmonary vasculature, which can be caused by or result in vascular remodeling and stiffening of the pulmonary artery [9, 10]. While some cases of PH are the end result of left-sided heart disease that directly influence systemic circulation (World Health Organization (WHO) functional group 2) [11], pulmonary arterial hypertension (PAH; WHO functional group 1) is the result of conditions, such as connective tissue diseases, acting directly on the pulmonary vasculature [12]. In these cases, although there is no initial left heart dysfunction, right ventricular pressure overload induced by PAH can lead to a shift or distortion of the interventricular septum [13–15]. Left heart dynamics in these patients may also be altered by reduced left ventricular (LV) preload as a result of inhibited right heart flow [16], increased vascular impedance from stiffening of the aorta [17], and co-existing systemic hypertension [18]. These factors may lead to a reduced LV volume in PAH patients relative to a healthy population, which in turn may lead to diminished stroke volume and cardiac output.

PH screening and assessment is clinically performed with echocardiography, which is limited in its capabilities due to its short penetration depth [19] and difficulty in properly aligning the acoustic window with the arterial flow [20]. In addition, Doppler ultrasound does not directly measure flow, but instead produces estimates from velocity measurements and pre-established vessel area models, which can introduce systematic errors into flow estimates [21].

Magnetic resonance imaging (MRI) provides the opportunity for more accurate visualization and quantification of flow via phase contrast (PC) acquisitions. MRI allows for less dependency on the skill of the operator as a result of improved spatial resolution, larger imaging windows, and higher tissue contrast. Recent work has also demonstrated that non-invasive measures of arterial stiffness are possible with PC MRI through metrics such as pulse wave velocity (PWV) and the relative area change (RAC) of the artery between diastole and systole [22].

Despite the advantages MRI could offer over existing exercise imaging modalities in assessing pulmonary hypertension, exercise MRI is challenging because of the relatively slow acquisition speed and the narrow confinements of the magnet bore. Instead, pharmacological stress agents, such as Dobutamine, Adenosine, and Regadenoson [23–25], are used in clinical practice. The drawback of such techniques is that pharmacological stress is not physiologic and, therefore, is not the preferred method of stress for assessing pulmonary hypertension [1, 26]. In response to these shortcomings, recent advances have seen the development of MRI-compatible exercise devices that allow for exercise in the MRI room adjacent to the table [27] or in a supine position on the scanner bed inside the magnet bore [28–30].

Overall, while it is likely that a patient diagnosed with PAH will experience diminished left heart function due to factors such as right ventricular pressure overload or reduced LV preload during the progression of the disease, such effects have not been investigated in detail with exercise MRI. Previous work has performed straightforward comparisons of blood flow in the aorta of PAH patients with controls using PC MRI [31], but has not correlated these measurements with metrics of vessel stiffness or investigated the value of exercise studies for these comparisons. The goal of this study was to use a custom-made, MRI-compatible exercise device to examine the effects of pulmonary arterial hypertension on aortic flow and stiffness in response to exercise with PC MRI. We hypothesize that PH subjects will show reduced left heart efficiency (lower stroke volume, higher heart rate) relative to healthy controls and these differences will be more pronounced following exercise stress.

## Methods

### Subject population

This study was approved by the local Institutional Review Board and was compliant with the Health Insurance Portability and Accountability Act. Twenty-one subjects were recruited, provided written informed consent, and were screened via an MRI safety questionnaire. The 21 subjects were divided into 3 cohorts: 7 young, healthy controls (28 ± 4 years; 3 male, 4 female), 8 older, healthy controls (58 ± 10 years; 1 male, 7 female), and 6 pulmonary hypertension patients (WHO Group 1 (PAH) from systemic sclerosis, 60 ± 9 years; 1 male, 5 female). All control subjects were free of overt cardiovascular, pulmonary, and renal disease. The recruited PH subjects had no recent syncope and, like the controls, had no history of unassociated lung or cardiovascular disease. Exclusion criteria for this study included classification as New York Heart Association functional class IV (symptoms of limited cardiac capacity apparent at rest) and skeletal or muscle abnormalities which could prohibit exercise. The older, healthy control group was age-matched to the PH group. The younger control group was included to provide insight regarding loss of cardiac function via disease versus natural loss due to aging. Subjects were excluded from the final statistical analysis if image quality was too poor to delineate the aortic wall or if the subject moved out of the prescribed scanning planes during exercise.

### MRI-compatible exercise device

**Fig. 1:**
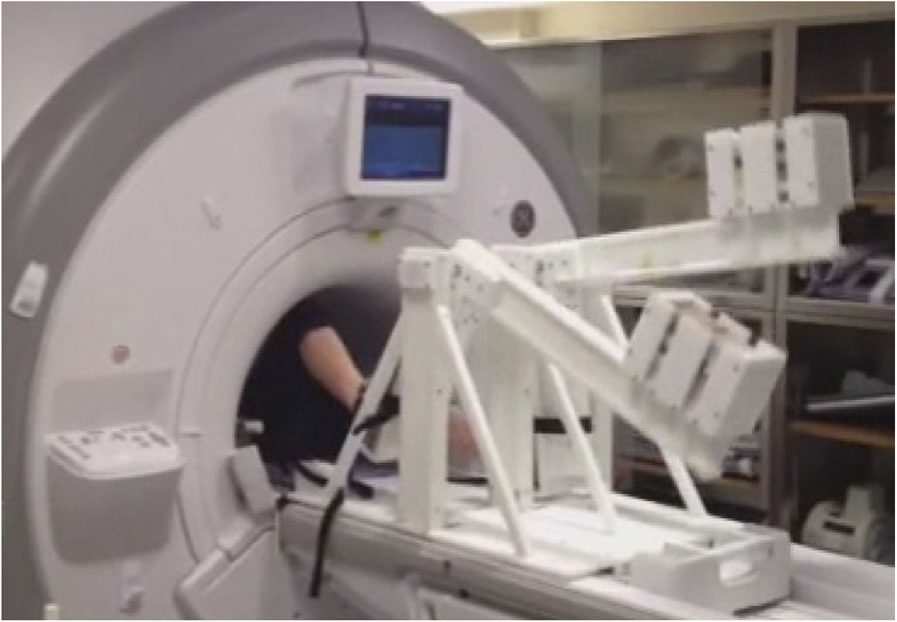
MRI-compatible exercise device. The subject exercises in the magnet bore via a dynamic stepping motion to the beat of a metronome. Resistance is controlled by removable weights at the end of each lever

Exercise was conducted with a custom-built, low-cost MRI-compatible stepping device which allowed subjects to exercise in a supine position in the scanner bore (Fig. 1) [28]. This permitted localizers and pre-scans to be performed prior to exercise, allowing for immediate image acquisition following the cessation of exercise. Imaging was performed following exercise rather than during exercise due to the challenging nature of breath holds during exercise, specifically in the PH population. Subjects exercised via a dynamic stepping motion to the beat of a metronome at the target exercise frequency. The exercise resistance was controlled by removable weights at the end of each lever arm. Optical displacement sensors on each lever arm provided the actual frequency of the stepping motion, allowing for real-time monitoring of the actual stepping frequency and power achieved by the subject relative to the targeted power level. To minimize axial displacement of the subject from the stepper as well as minimize chest motion during exercise, the subject was connected to the exercise equipment via a backpack harness and had hand-grips to hold on to for stabilization.

### Data acquisition

Imaging was performed on one of two clinical 1.5 T MRI systems (HDxt or Discovery 450w, GE Healthcare, Waukesha, WI) using an 8 channel cardiac coil and vector electrocardiographic (ECG) gating. Flow measurements were acquired with a prospectively ECG-gated two-dimensional (2D) cine phase contrast (PC) sequence performed across a 15 s breath hold. The imaging plane was prescribed orthogonal to the ascending aorta. Imaging parameters for the PC sequence were: field of view = 35 × 35 cm, matrix size = 128 × 256 (reconstructed to 256 × 256), slice thickness = 8 mm; TR/TE = 6.1/3.7 ms, flip angle = 30°, velocity encode (VENC) = 150 cm/s, parallel-imaging (ASSET) acceleration factor = 2, temporal resolution = 25 ms, and cardiac phases = 20.

**Fig. 2:**
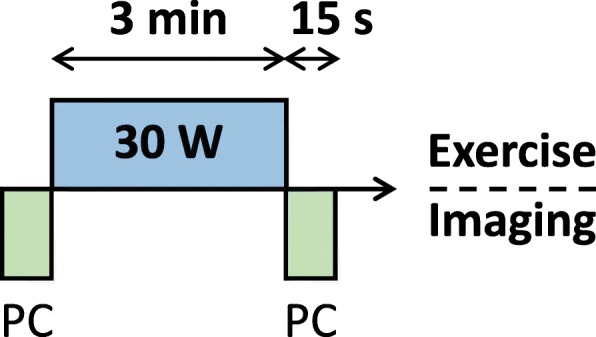
Exercise and imaging paradigm for all three groups. PC imaging was performed in the ascending aorta at rest prior to exercise. This was followed by 3 min of exercise at a power of 30 W. An identical PC scan to that performed at rest immediately followed the end of exercise

All subjects exercised at a low exercise power to guarantee the PH subjects would be able to complete the same exercise paradigm as the control groups. This exercise and imaging paradigm was designed to induce mild-to-moderate exercise stress in all subject groups (Fig. 2). A PC acquisition was performed at rest prior to exercise. Subjects then exercised at a target power of 30 W for 3 min. Immediately following the end of exercise, subjects held their breath for a second PC MRI acquisition in the ascending aorta. Total examination time was less than an hour, including set-up and take-down of the exercise equipment. Subjects typically spent less than 30 min in the scanner bore.

### Data analysis

Aortic flow measurements were performed with CV Flow (Version 3.3, Medis, Leiden, Netherlands) on a GE Advantage workstation. The following parameters were calculated from the flow measurements: stroke volume (SV), cardiac output (CO), peak systolic flow (PSF), peak systolic velocity (PSV), and the relative area change (RAC) of the ascending aorta between diastole and systole. Aortic stiffness was estimated with relative area change rather than pulse wave velocity due to the limited temporal resolution of the PC sequence.

### Statistical methods

All statistical analysis was performed with R (Version 3.2.3, R Foundation for Statistical Computing, Vienna, Austria). Results are presented as the mean value plus or minus one standard deviation of the sampled group. To determine the statistical significance of any intragroup changes between parameters measured at rest and stress, a paired student’s t-test was performed for each cohort. A one-way ANOVA test with a post-hoc Tukey’s honestly significant difference (HSD) test was used to determine statistically significant intergroup differences for each parameter at rest and stress. Levene’s test was used in conjunction with the ANOVA test to ensure that the assumption of equal variances between groups was valid. For all statistical tests, a threshold of α = 0.05 was chosen for statistical significance.

## Results

MRI exercise stress tests were successfully performed in 19/21 subjects. Two of the older, healthy controls shifted their body position during exercise such that the prescribed scan planes were not usable. Thus, they were omitted from the analysis. All subjects successfully completed the 15 s breath hold following exercise. Although the target exercise power for each group was 30 W, the actual exercise power achieved for the remaining young controls, older controls, and PH subjects was 38 ± 12 W, 32 ± 8 W, and 30 ± 10 W respectively. There was no statistically significant difference in the achieved exercise power between these groups (*p* = 0.29). These exercise powers resulted in an average heart rate increase of 46% for the young controls, 29% for the older controls, and 74% for the PH subjects.

**Fig. 3:**
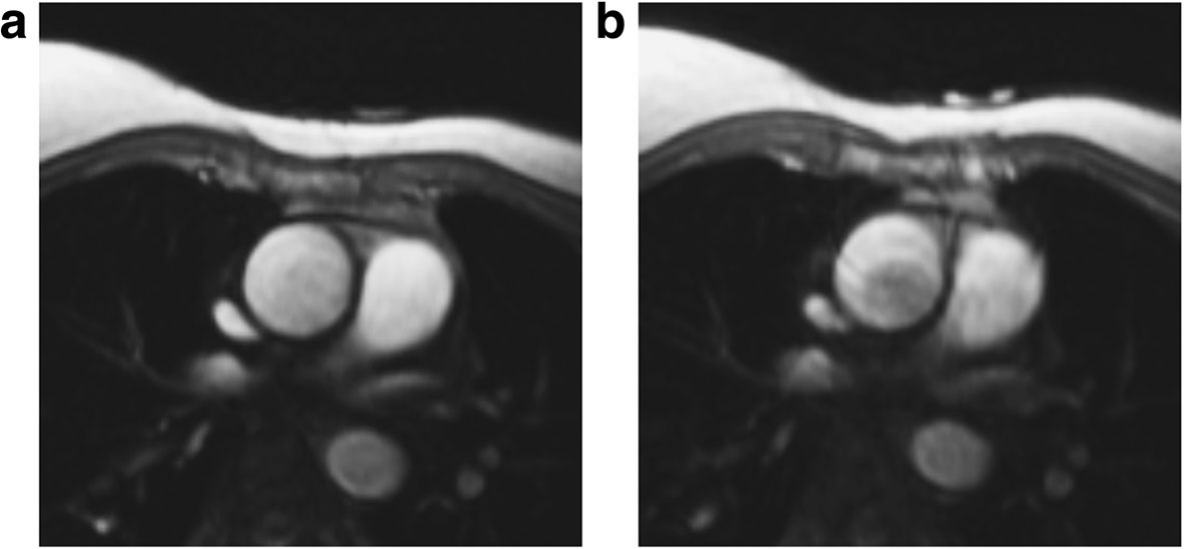
Representative magnitude PC image quality in the ascending aorta (**a**) at rest and (**b**) immediately following exercise. Mild blurring was observed in images following exercise alongside some darkening of the blood, but image quality was still sufficient to delineate relevant vasculature

Figure 3 shows a representative comparison of image quality in magnitude PC images before and immediately following exercise. For most subjects, only minimal motion artifacts were present following exercise, allowing for good segmentation of the aorta. Minor blurring was observed in the post-exercise images and reduced blood signal was seen in some areas of the aorta. Increased blurring was observed in the PH subjects relative to the control groups.

**Fig. 4:**
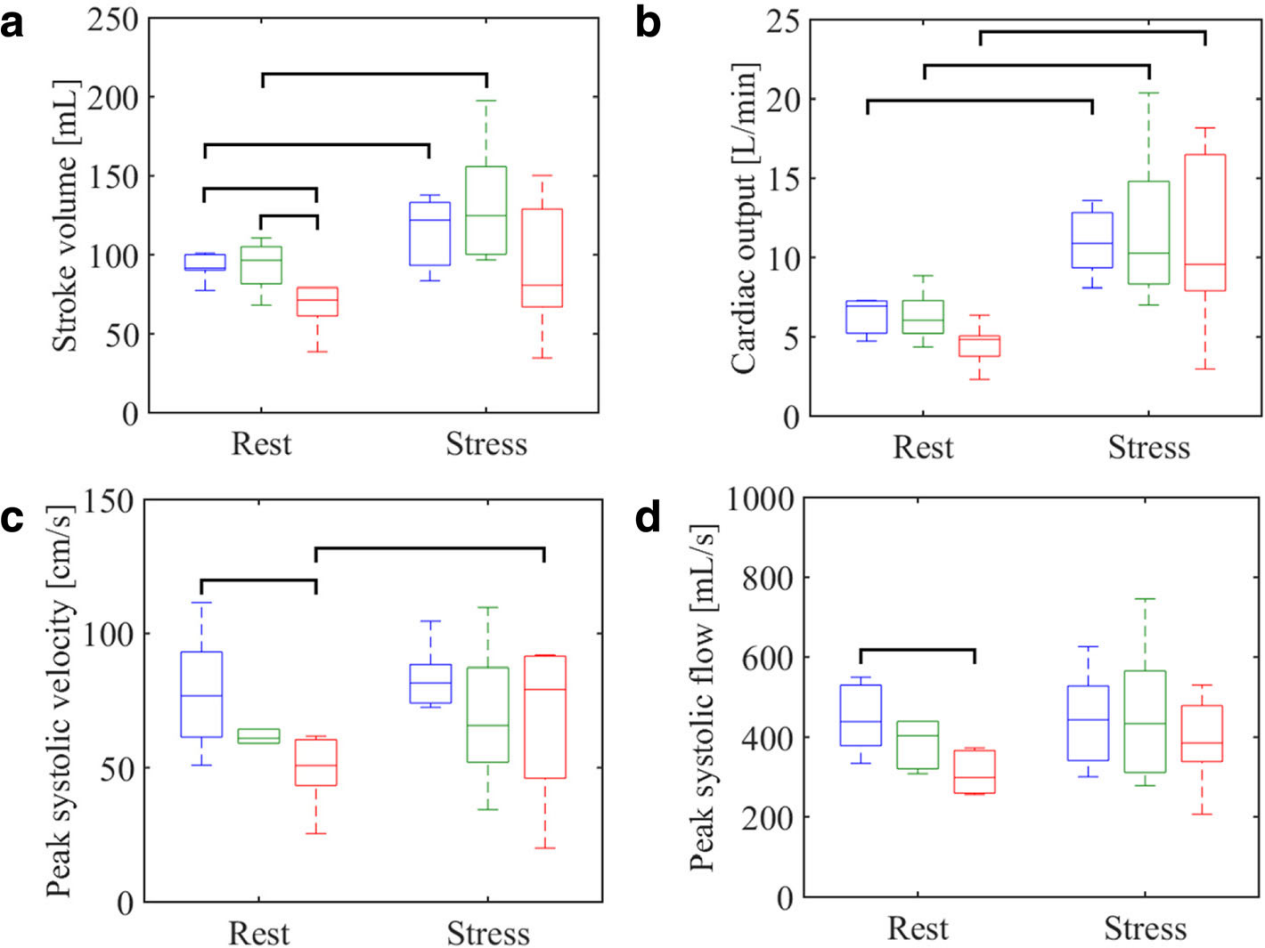
Boxplots displaying the distribution of key aortic flow variables as measured with PC MRI in the ascending aorta: (**a**) stroke volume, (**b**) cardiac output, (**c**) peak systolic velocity, and (**d**) peak systolic flow. Blue indicates young controls, green indicates older controls, and red represents PH subjects. A bracket between two boxplots indicates a statistically significant (*p* < 0.05) difference between the two groups of measurements

Table 1 presents the average values plus/minus one standard deviation for each hemodynamic parameter measured from the PC scans. The data distributions for SV, CO, PSV, and PSF are visualized at rest and following stress for all cohorts in Fig. 4. Statistically significant increases in heart rate, stroke volume, and cardiac output following exercise, indicated that the mild exercise paradigm was able to induce a cardiac response in all cohorts. On average, stroke volumes in the PH subjects were 30% lower than the young controls and 28% lower than the older controls at rest, and 23% lower than the younger controls and 32% lower than the older controls following exercise. Interestingly, however, PH subjects demonstrated a resting cardiac output approximately 30% lower than their healthy counterparts, while mean cardiac output measurements were comparable amongst all groups following exercise (Fig. 4b).

**Table 1:** Hemodynamic parameters measured in the ascending aorta at rest and immediately following exercise stress from phase contrast acquisitions. Results are presented as the mean value plus or minus one standard deviation of the samples

Variable	Rest			Stress		
	Young	Older	PAH	Young	Older	PAH
Heart rate [bpm]	66 ± 11	67 ± 9	69 ± 19	97 ± 19*	87 ± 12*	116 ± 19*
Stroke volume [mL]	96 ± 15	93 ± 16	67 ± 16	116 ± 23*	133 ± 40*	90 ± 42
Cardiac output [L/min]	6.3 ± 1.1	6.3 ± 1.6	4.5 ± 1.4	11 ± 2*	11.8 ± 5*	10.8 ± 5.7*
Peak systolic velocity [cm/s]	77 ± 21	58 ± 15	49 ± 14	78 ± 22	69 ± 27	68 ± 29*
Peak systolic flow [mL/s]	445 ± 85	416 ± 114	309 ± 52	446 ± 118	462 ± 176	388 ± 113
Relative area change	0.28 ± 0.08	0.17 ± 0.05	0.15 ± 0.05	0.28 ± 0.10	0.27 ± 0.14	0.25 ± 0.18

*Denotes statistical significance (*p* < 0.05) relative to the same cohort at rest

Measurements of peak systolic flow and peak systolic velocity showed no meaningful response to exercise in the young control groups. In contrast, the older controls showed a 19% increase in velocity and an 11% increase in flow in response to exercise. Likewise, the PH subjects demonstrated a larger 39% increase in velocity and 26% increase in flow. At both rest and exercise, the PH subjects demonstrated lower overall average values of these parameters when compared to both control groups (Fig. 4c, d).

**Fig. 5:**
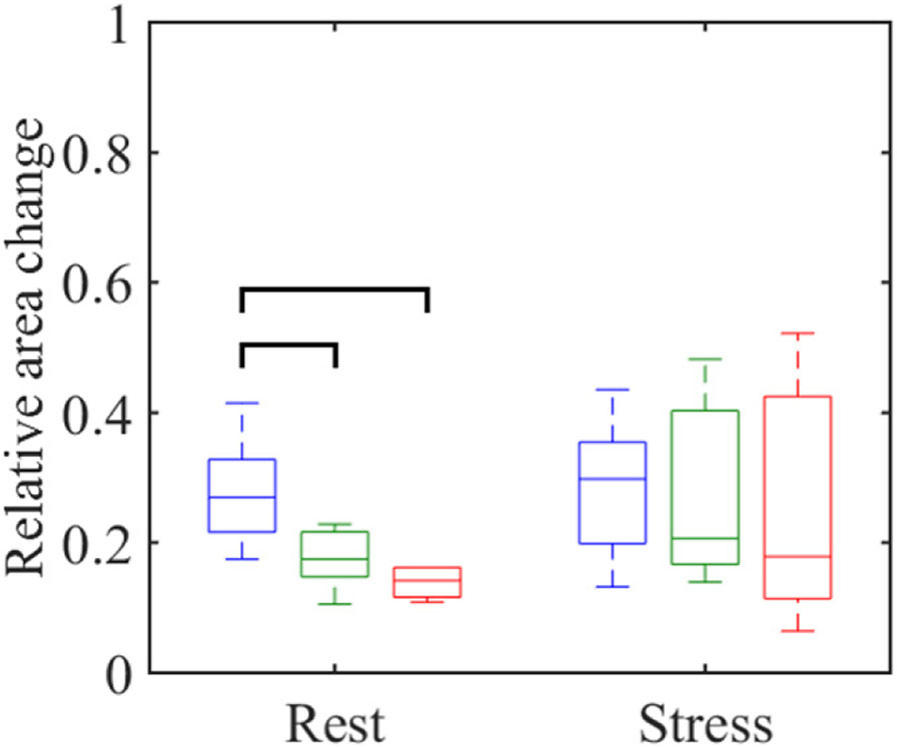
Distribution of aortic wall stiffness as calculated with the relative area change method in the ascending aorta. Blue indicates young controls, green indicates older controls, and red represents PH subjects. A bracket between two boxplots indicates a statistically significant (*p* < 0.05) difference between the two groups of measurements

The distribution of aortic wall stiffness measurements for each cohort is presented in Fig. 5. Significantly lower values of RAC in the older controls and PH subjects relative to the young controls at rest indicated stiffer aortic walls in these groups. Furthermore, the PH subjects had the lowest mean RAC of all groups, suggesting the stiffest vessel walls amongst the three cohorts. Although not significant, exercise showed increased vessel compliance of the older controls and PH subjects to the level of the young controls. Overall, measurements for all hemodynamic parameters following exercise usually showed more variance amongst each cohort than at rest.

## Discussion

As hypothesized, PH patients demonstrated reduced LV stroke volume at rest and during stress when compared to control groups. These reduced stroke volumes may suggest the presence of a shifted interventricular septum induced by right ventricular pressure overload, reduced LV preload, or a co-existing condition, such as systemic hypertension. Future studies with imaging sequences dedicated to ventricular imaging (e.g. balanced steady-state free precession MRI) and ventricular pressure measurements could prove valuable to elucidate the relative contributions of these factors to the reduced left heart SV in these patients. Despite low stroke volumes, however, the PH patients presented a preserved cardiac output following exercise as a result of a compensatory increase in heart rate (74% increase in PH subjects as compared to 29% in age-matched controls). Therefore, while PH patients may be able to match exercise capabilities of age-matched controls in the short-term, the more rapid increase in heart rate will result in PH patients reaching a clinically-defined maximum heart rate sooner, yielding reduced exercise endurance. This behavior in the left ventricle is consistent with right heart dynamics previously reported following exercise in patients with PH [32].

Although the exercise stress induced comparable levels of cardiac output in the PH population and both control groups, the PH subjects demonstrated reduced peak systolic velocity and peak systolic flow – further evidence of altered left heart hemodynamics induced by right heart changes from PAH. It is worth noting, however, that for these parameters the older control group showed decreased mean values relative to the younger group as well, suggesting the reduced capacity shown in the PH subjects may be a more complex dynamic between natural age-related loss of cardiovascular function and disease progression.

The relative area change of the aorta proved to be capable of assessing vessel stiffness, demonstrating reduced aortic compliance in the PH subjects relative to the controls. This result was consistent with the fact that the recruited patients developed PAH as a result of ongoing systemic sclerosis [33] and may have contributed to the reduced stroke volume and exercise capacity in this group. There is great value in future investigations to perform non-invasive measures in the pulmonary artery of the PH subjects to examine the extent of stiffening relative to the aorta.

The modest increase in heart rate observed across all three subject cohorts validated that the relatively low exercise power used in this study was still effective at inducing observable exercise stress in each cohort, although the cardiac response was diminished in the control groups. This demonstrated that mild exercise paradigms can be an effective method for stress imaging in subjects with reduced cardiac capacity. The drawback of using low exercise powers was observed in the younger controls, however, who were not as challenged by the paradigm and, on average, exercised at a power 25% above the targeted level. Since the resistance could be adjusted in real-time during exercise with the exercise equipment used for this study, in this case, where the young controls exercised faster than this metronome beat, there was no way to compensate and reduce their exercise power.

The potential value of performing exercise studies in a PH population was apparent, as the PH cohort showed larger relative responses to exercise than the healthy controls in all measured hemodynamic parameters except stroke volume, which is known to have a reduced response to exercise in PH subjects [32]. Performing studies with increased exercise power may induce greater differences between these cohorts than observed with the mild exercise powers used in this study. The increased cardiac response to higher exercise powers in all cohorts would also likely result in statistically significant differences between rest and stress in many of the parameters that showed non-significant increases with exercise, such as peak systolic velocity and flow. Given the disparity in exercise abilities between these cohorts, in future studies, it would be valuable to standardize our comparisons on more physiologic measures of exercise, such as a common heart rate or maximal oxygen uptake (VO_2,max_), rather than a common exercise power.

While the breath held PC acquisitions were successfully completed in all subjects following exercise, some of the subjects with more limited cardiac output, expressed a greater perceived difficulty in accomplishing these breath holds. Body motion from discomfort during these breath holds may have contributed to the larger degree of blurring observed in these subjects’ images. Although more challenging for these subjects, it was essential to begin imaging immediately following exercise due to the rapid recovery of heart rate back to a resting state. The potential heart rate recovery across the 15 s of imaging following cessation of exercise was not deemed to be a concern, however, as a previous study showed that even in young athletes, heart rate will only decrease an average of 5% in this time period [34]. In future studies, the implementation of free-breathing or real-time acquisitions could eliminate the need for a breath hold altogether and allow for imaging during exercise, resulting in a more comfortable exercise paradigm for subjects and a possible improvement in image quality.

There were a few limitations to this study beyond those already mentioned. The harness used to reduce chest motion was not entirely effective at preventing subject movement, as demonstrated with the two subjects who moved out of their prescribed scan planes. Motion such as this possibly led to shifts or rotations in scan planes in the other subjects, which could impact the accuracy of flow and vessel cross-sectional area measurements. In addition, the statistical power of our observations was limited by the relatively small sample size of each cohort. These factors likely contributed to increased variance and lack of statistical significance in some of the measurements.

## Conclusion

In conclusion, we investigated the impact of pulmonary arterial hypertension on aortic flow with PC MRI following a modest exercise challenge. Custom-made, MR-compatible exercise equipment permitted subjects to exercise in a supine position fully in the bore of the scanner, allowing for imaging immediately following the cessation of exercise. As we hypothesized, the PH subjects showed reduced LV stroke volume and decreased cardiac efficiency relative to the healthy controls. The PH subjects demonstrated a different mechanism for increasing cardiac output in response to exercise, relying on large increases in heart rate rather than the balanced increase in heart rate and stroke volume observed in healthy controls. Cardiac output, peak systolic velocity, and peak systolic flow showed increased sensitivity to exercise-induced changes in the PH population, as well. Future studies will compare these metrics to those obtained from pulmonary flow in the same subject populations.

## Abbreviations

CO: Cardiac output
LV: Left ventricle
MRI: Magnetic resonance imaging
PAH: Pulmonary arterial hypertension
PC: Phase contrast
PH: Pulmonary hypertension
PSF: Peak systolic flow
PSV: Peak systolic velocity
PWV: Pulse wave velocity
RAC: Relative area change
SPECT: Single photon emission computer tomography
SV: Stroke volume
VENC: Velocity encoding
WHO: World Health Organization
